# The gender peak effect: Women are most vulnerable to infections during COVID-19 peaks

**DOI:** 10.3389/fpubh.2022.937179

**Published:** 2022-08-09

**Authors:** Cary Wu, Yue Qian

**Affiliations:** ^1^Department of Sociology, Faculty of Liberal Arts and Professional Studies, York University, Toronto, ON, Canada; ^2^Department of Sociology, Faculty of Arts, University of British Columbia, Vancouver, BC, Canada

**Keywords:** gender, COVID-19 peaks, care work, Canada, infection

## Abstract

In this article, we describe a gender peak effect that women's relative share in COVID-19 infections increases when there is a sharp increase in cases, and it reaches the highest level during peak times in each wave of the COVID-19 outbreak. We demonstrate this gender peak effect by analyzing detailed, sex-disaggregated Public Health Agency of Canada (PHAC) data. The data include 1,045,998 men and women who were confirmed cases of COVID-19 from March 2020 to February 2022. We show that women's relative share in COVID-19 infections always increases and reaches the level exceeding men's share when we see a sharp peak in case number. We further show that women's higher share in care work (e.g., captured by occupation and age variables) largely explains their elevated infections during COVID-19 peaks. Effective public health interventions during infectious disease outbreaks must recognize this potential gender peak effect and take appropriate measures to curb women's health vulnerabilities.

## Introduction

Success in public health requires effective and equitable responses to disease outbreaks. A fundamental key to achieving such responses lies in pinpointing how people are unequally affected (1). In particular, women and men often fare differently in disease outbreaks. This is due to both biological sex features and socially constructed gendered responsibilities (2). The present COVID-19 pandemic is no exception (1, 3). A consistent pattern documented in almost all countries with sex-disaggregated data is that, if infected with COVID-19, men experience a higher risk of severe illness and death, compared with similarly-aged women (3–9). Scholars suggest that men's higher severity and mortality of COVID-19 are more likely due to biological sex differences (e.g., sex-based immune responses), although gender differences in health behaviors (e.g., smoking) and pre-existing conditions (e.g., diabetes, hypertension) could also play an important role (1, 3, 4, 8, 10).

By contrast, research on the overall difference in COVID-19 infections between men and women has reported mixed findings (5, 8, 9, 11–13). Still, two specific empirical findings seem consistent. First, differences in infection rates are age-dependent: women show higher infection rates than men in prime working ages but the reverse is true in young or retirement ages (14). Second, women's higher representation in health- and care-related occupations explains a large portion of the differences in infection rates between women and men (5, 14). These findings suggest that the differences in susceptibility to COVID-19 infection between men and women are primarily a result of gendered work-family responsibilities that place women at the forefront of the pandemic (1, 3, 5).

Two years into the COVID-19 pandemic, there have been ebbs and flows of cases, but little is known about how gendered health impacts vary over the course of the pandemic. In this study, we seek to demonstrate that gender differences in infection rates are also time-dependent. Most notably, when there is a sharp increase in COVID-19 cases, women's infection rate increases disproportionately to exceed that of men.

There are two main reasons why women are most vulnerable to infection during COVID-19 peaks. First, given the infectious nature of COVID-19, women's predominant roles as caregivers within families and as frontline health care and community workers expose them to a high risk of infection (1, 3, 5). In the workforce of almost all countries, women represent the majority of frontline workers in health care and other essential high-contact jobs (2, 15). At home, women shoulder the majority of care work, including caring for not only children but also sick family members (3, 16). Caregiving demands from families and workplaces are likely intensified during COVID-19 peaks, thereby leading to a greater infection risk for women than for men.

Second, disease outbreaks, especially during peak times, often exacerbate pre-existing gender inequalities, which in turn exposes women to a high risk of infection (17). Pre-existing gender inequalities include, for example, women's disadvantage relative to men in access to support services, health care, medical treatments, and economic opportunities, which are often amplified during disruptive times when resources are much scarcer (18). Pre-existing occupational gender segregation and insufficient financial resources may compel women essential workers to continue performing on-site, high-contact jobs (15), which places them at elevated risk of infection especially when COVID-19 cases are rapidly rising. On the policy level, women's underrepresentation in leadership positions means that they wield little influence over the decision-making on outbreak responses (1, 19). As a result, women's needs are largely unmet (18), leaving them more vulnerable than men in times of peaked turbulence.

Taken together, we expect that, across major waves of outbreak in the pandemic, the share of women in COVID-19 infections increases as the number of confirmed cases rises, and it likely peaks when case number peaks. Further, we expect that women's higher share in care work largely explains their elevated infections during peak times.

## Methods

We use detailed Public Health Agency of Canada (PHAC) data on confirmed cases of COVID-19. The current dataset (Release date: 11-02-2022) records a total of 1,048,575 people in Canada who tested positive for COVID-19 from 15 January of 2020 to 8 February of 2022. The dataset is a subset of the total counts reported by the health authorities across Canada since it only accounts for those where a detailed case report was provided by the provincial or territorial jurisdiction to the PHAC. The data include information on each confirmed case's episode time (week and year) and demographic characteristics such as gender, occupation, and age group. The 1,048,575 cases consist of 500,526 men (47.7%), 545,472 women (52.2%), and 2,577 case with gender “not stated” (0.25%). Our analyses in this article only include confirmed cases of women and men. Population estimates from Statistics Canada report that the resident population of Canada was 38,246,108 including 18,238,276 women (50.3%) and 19,007,832 men (49.7%), as of September 2021 (Statistics Canada 2021). Table 1 provides the summary statistics of key variables, overall and by gender groups.

**Table 1:** Summary statistics of key variables in analysis overall and by gender groups.

	**Overall** **(*n* = 1,048,575)**	**Men** **(*n* = 500,526)**	**Women** **(*n* = 545,472)**
**Episode year**			
2020	0.211	0.213	0.209
2021	0.593	0.611	0.577
2022	0.193	0.172	0.211
Not stated	0.003	0.003	0.003
***Region***			
Atlantic	0.011	0.011	0.011
Quebec	0.285	0.277	0.294
Ontario and Nunavut	0.350	0.351	0.347
Prairies the Northwest Territories	0.245	0.250	0.241
British Columbia and Yukon	0.109	0.112	0.106
***Age group***			
0 to 19 years	0.203	0.216	0.191
20 to 29 years	0.195	0.193	0.197
30 to 39 years	0.175	0.171	0.179
40 to 49 years	0.150	0.144	0.156
50 to 59 years	0.123	0.124	0.121
60 to 69 years	0.074	0.079	0.069
70 to 79 years	0.038	0.040	0.036
80 years or older	0.042	0.033	0.051
Not stated	0.001	0.001	0.000
***Occupation***			
Health care worker	0.061	0.025	0.093
School or daycare worker/attendee	0.010	0.004	0.016
Long term care resident	0.007	0.005	0.008
Other	0.512	0.542	0.486
Not stated	0.410	0.423	0.397
***Hospitalization status***			
Hospitalized and in intensive care unit	0.007	0.009	0.005
Hospitalized, but not in intensive care unit	0.034	0.036	0.031
Not hospitalized	0.645	0.643	0.649
Not stated/Unknown	0.314	0.311	0.314
***Death***			
Yes	0.011	0.012	0.010
No	0.932	0.937	0.927
Not stated	0.057	0.051	0.062

## Results

**Figure 1:**
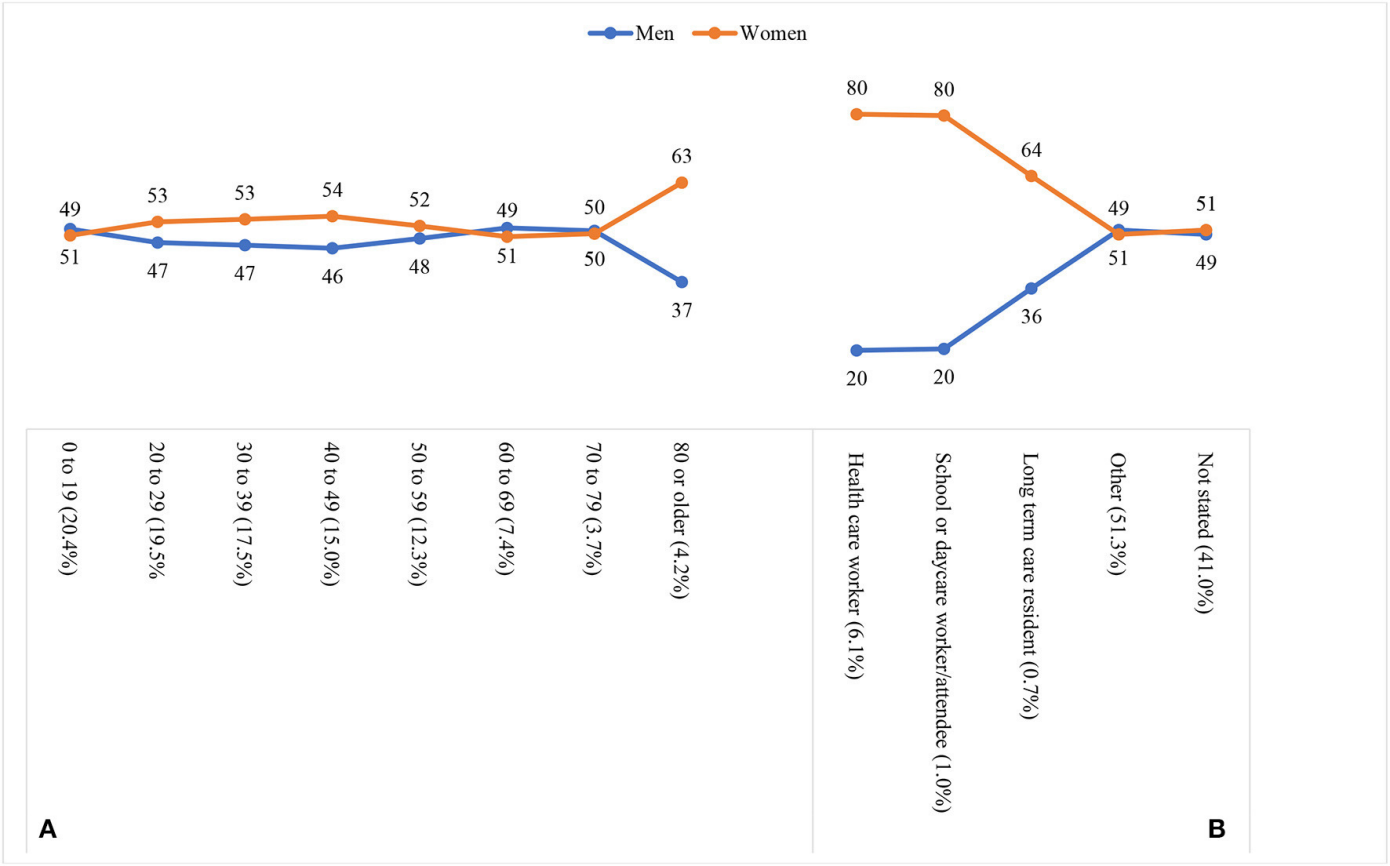
Gender distribution of COVID-19 infections by age group **(A)** and occupation **(B)**. The share of cases in each age group or occupation is indicated by the percentage in parentheses.

Figure 1 visualizes the gender distribution of COVID-19 infections by age group (Figure 1A) and occupation (Figure 1B). Two empirical patterns are clear. First, gender differences in infections are age-dependent. Among the prime working-age population (20–59 years), women account for a higher share of confirmed COVID-19 cases (i.e., infections) than men, whereas the reverse is true in younger age groups (0–19 years) and older age groups (60–69 years). Women's higher share in the age group of 80 or older is likely due to their longer life expectancy (14). Second, gender differences in infections change across occupational categories. The share of women in confirmed cases is much higher among health care workers, school or daycare workers/attendees, and long-term care residents, whereas in “other” and “not stated” occupational categories, women and men share equal representation. These patterns are consistent with previous research (14).

**Figure 2:**
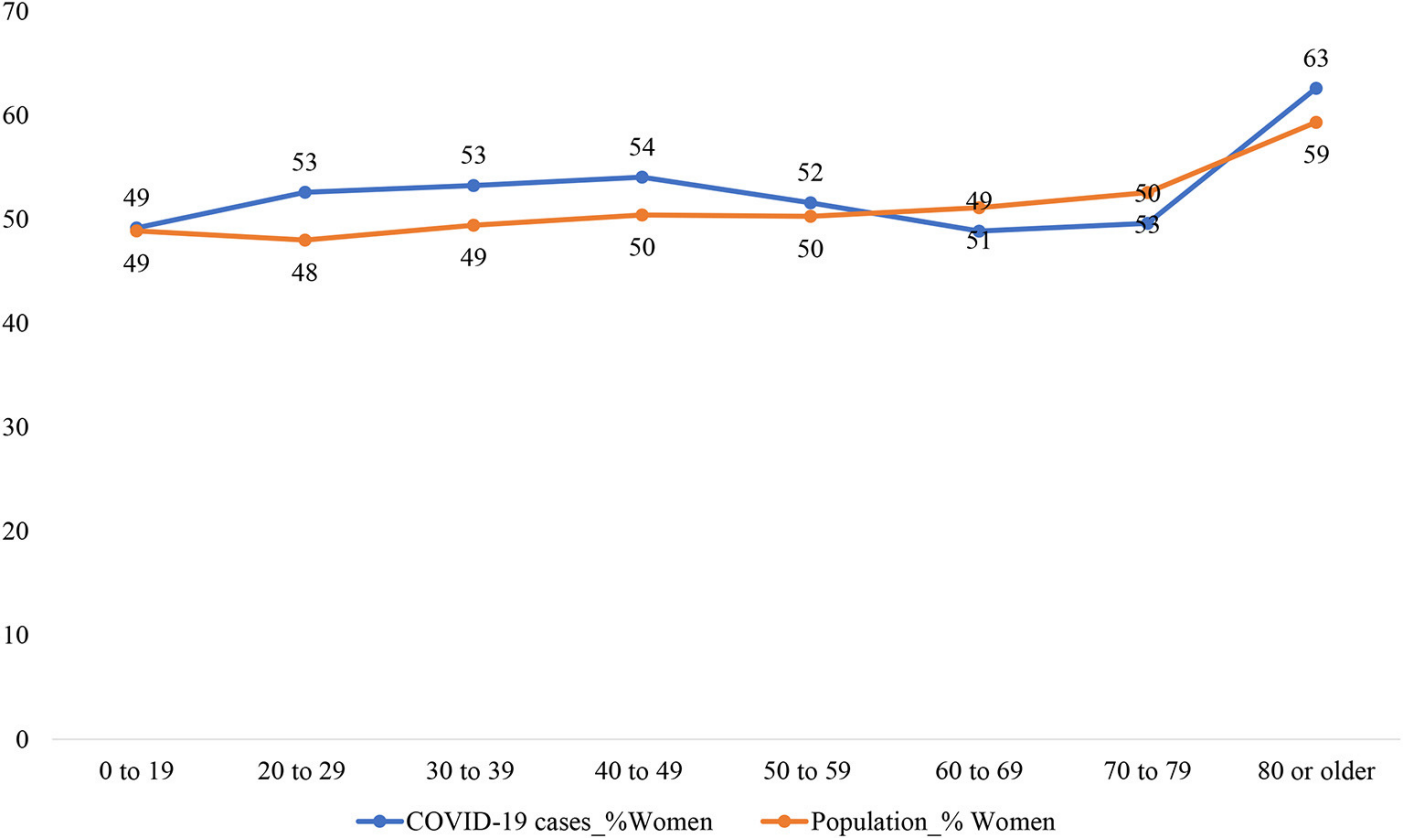
Comparing the share of women among COVID-19 cases and among the general population. The share of women among the general population is estimated using data from Statistics Canada (2021): doi: 10.25318/1710000501-eng.

To provide further support that working-age women are particularly vulnerable, Figure 2 compares the share of women among COVID-19 cases and the share of women among the general population across age groups. Among the young age group (0–19), the share of women among COVID-19 cases is identical to the share of women among the general population. This changes among the prime working-age groups (20–59 years): the share of women is about 4–5 percent point higher among the COVID-19 cases than among the general population. In older age groups (60–79 years), the share of women among infected cases becomes 2–3 percent point lower than the share among the general population. Again, women's higher share in the age group of 80 or older is likely due to their longer life expectancy.

**Figure 3:**
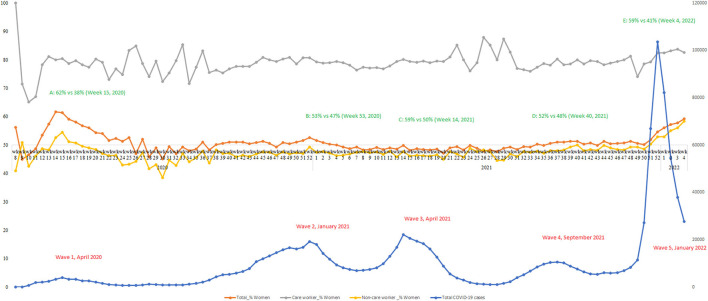
Gender trajectories of relative COVID-19 infections in Canada, 2020–2022.

Finally, Figure 3 depicts the number of confirmed COVID-19 cases (blue line), the percentage of women in the overall confirmed cases (orange line), and the percentage of women in confirmed cases among care workers (including health care workers and school or daycare workers/attendees; gray line) as well as among non-care workers (amber line), from March 2020 (2020 week 8) to February 2022 (2022 week 4). Since the COVID-19 pandemic started in March 2020, Canada has been hit by five major waves including, roughly, the first wave from March to July 2020 (peaks in April 2020), the second wave from August 2020 to February 2021 (peaks in January 2021), the third wave from March to July 2021 (peaks April 2021), the fourth wave from August to November 2021 (peaks in September 2021), and the most recent fifth wave from December 2021 to February 2022 (peaks in January 2022). Clearly, the share of women in COVID-19 infections (orange line) always showed an increase as the number of confirmed cases increased in each wave, and women's infections relative to men peaked (i.e., A–E) during each wave's peak time. One concern is that the gender pattern could come from the gender difference in vaccination rate. Our data do not include information about vaccination status for each individual. However, data from Government of Canada website on vaccination coverage show that overall women have higher vaccination rates than men. This is especially true among working age groups.

Further, to demonstrate that women's higher share in care work can largely explain women's elevated infections during peak times, we separate and compare the changes in the percentage of women in infections among care workers (gray line) and non-care workers (amber line). Among infections in care workers, women accounted for the vast majority of cases (80%). The high percentage of women in infections among care workers was relatively stable during COVID-19 peaks, and it showed more fluctuations when the number of total cases was relatively low. Among infections in non-care workers, women's share was mostly lower than men's over the course of the pandemic (reference line y-axis = 50%). Still, we see clearly that women's share often reached the highest point during COVID-19 peaks in each wave. These findings illustrate that women's higher share in care work (including work in health care and schools or daycare centers) largely explains women's elevated infections during peak times of the COVID-19 outbreak.

Taken together, our analysis yields two major findings. First, we show that the female-to-male infection ratio has always been higher during COVID-19 peaks as compared to non-peak times. Second, we show that women's higher representation in care work is likely the cause for the gender peak effect. It is important to note that our conclusion is based on our analysis of the *longitudinal* patterns in gender difference in infections, rather on gender difference in infections *per se*. In other words, we compare changes in gender difference in infections during COVID-19 peaks and non-peak times. Many factors could create gender differences in infection rate. But these factors are unlikely to change during a short period, and therefore they are not the cause for the time-dependent gender patterns. For this reason, we have argued that the time-dependent gender patterns are likely a result of women's higher representation in care work that makes women, working-age women in particular, especially vulnerable to infections during COVID-19 peaks.

## Discussions and public health implications

Infectious diseases that can be transmitted through human contact are occurring more often now than ever. Recent outbreaks include the 2002–2004 SARS, the 2013–2016 Ebola, the 2015 Zika virus, and the ongoing COVID-19 pandemic. Underlying the emergence of these outbreaks are global changes such as population growth, urbanization, climate change, and the increase in international travel and human connectivity (20). These changing global dynamics likely make future outbreaks even more lethal. For this reason, the World Economic Forum's 2020 Global Risks Report has listed infectious diseases as one of the top 10 risks in terms of impact for the next decade (21).

A key lesson from these outbreaks is that success in global public health requires responding to disease outbreaks effectively and equitably (17). Because of biological sex differences and societal gender inequalities, scholars have called for attention to understanding and responding to the gendered impacts of COVID-19 outbreaks since the very beginning of the pandemic (1). Previous research on gender differences in COVID-19 infections has focused on the role of age and occupation in shaping gendered patterns of infections (5, 14). Despite the evolving nature of infectious disease outbreaks, few studies have considered the time dimension of gendered health impacts over the course of the pandemic, a gap that we have filled in this study.

In this study, we have shown that gender differences in infections during COVID-19 outbreaks are time-dependent. When there is a sharp peak in COVID-19 cases, the share of women in COVID-19 infections always increases to a level exceeding the share of men. We have also revealed that women's higher share in care work largely explains their elevated infections during peak times. These findings suggest that women's predominant roles as caregivers in families and workforces expose them to a high risk of infection during COVID-19 peaks. Pre-existing gender inequalities in financial resources, access to health care, and decision-making power in the policy realms may further disadvantage women in times of rising infections (18).

Our finding calls for attention to the particular vulnerability that women experience during the peak times of COVID-19 and potentially future infectious disease outbreaks. When understanding differences in susceptibility to disease infection across segments of the population, time is a critical dimension because disease outbreaks usually last an extended period and different new variants likely emerge to increase the spread of the virus. When a disease outbreak occurs, researchers and policymakers should monitor how gender disparities change at different stages of the outbreak, and design response policies accordingly. Including gender and sex dimensions in public responses will help not only ensure effective and equitable responses but also minimize the chances that disease outbreaks reproduce or exacerbate gender inequalities (1, 18, 22).

## Data availability statement

Publicly available datasets were analyzed in this study. This data can be found at: https://www150.statcan.gc.ca/n1/en/catalogue/13260003.

## Author contributions

Both authors listed have made a substantial, direct, and intellectual contribution to the work and approved it for publication.

## Funding

Funding provided by the Canadian Institutes of Health Research (CIHR, CW, FRN-170368 and YQ, OV7-170372).

## Conflict of interest

The authors declare that the research was conducted in the absence of any commercial or financial relationships that could be construed as a potential conflict of interest.

## Publisher's note

All claims expressed in this article are solely those of the authors and do not necessarily represent those of their affiliated organizations, or those of the publisher, the editors and the reviewers. Any product that may be evaluated in this article, or claim that may be made by its manufacturer, is not guaranteed or endorsed by the publisher.
